# Pathway-based analysis using reduced gene subsets in genome-wide association studies

**DOI:** 10.1186/1471-2105-12-17

**Published:** 2011-01-12

**Authors:** Jingyuan Zhao, Simone Gupta, Mark Seielstad, Jianjun Liu, Anbupalam Thalamuthu

**Affiliations:** 1 Human Genetics, 60 Biopolis Street 02-01, Genome Institute of Singapore, 138672, Singapore; 2 McKusick - Nathans Institute of Genetic Medicine, School of Medicine, Johns Hopkins University, 733 N. Broadway St. Baltimore, MD 21205, USA; 3 Institute for Human Genetics, 513 Parnassus Avenue, University of California, San Francisco, CA 94143 and Blood Systems Research Institute, 270 Masonic Avenue, San Francisco, CA 94118, USA

## Abstract

**Background:**

Single Nucleotide Polymorphism (SNP) analysis only captures a small proportion of associated genetic variants in Genome-Wide Association Studies (GWAS) partly due to small marginal effects. Pathway level analysis incorporating prior biological information offers another way to analyze GWAS's of complex diseases, and promises to reveal the mechanisms leading to complex diseases. Biologically defined pathways are typically comprised of numerous genes. If only a subset of genes in the pathways is associated with disease then a joint analysis including all individual genes would result in a loss of power. To address this issue, we propose a pathway-based method that allows us to test for joint effects by using a pre-selected gene subset. In the proposed approach, each gene is considered as the basic unit, which reduces the number of genetic variants considered and hence reduces the degrees of freedom in the joint analysis. The proposed approach also can be used to investigate the joint effect of several genes in a candidate gene study.

**Results:**

We applied this new method to a published GWAS of psoriasis and identified 6 biologically plausible pathways, after adjustment for multiple testing. The pathways identified in our analysis overlap with those reported in previous studies. Further, using simulations across a range of gene numbers and effect sizes, we demonstrate that the proposed approach enjoys higher power than several other approaches to detect associated pathways.

**Conclusions:**

The proposed method could increase the power to discover susceptibility pathways and to identify associated genes using GWAS. In our analysis of genome-wide psoriasis data, we have identified a number of relevant pathways for psoriasis.

## Background

Genetic association studies aim to detect associations between disease phenotypes and genetic variants. A commonly used tool to establish association between a SNP and a disease is to perform statistical tests of association for each individual SNP marker. A multiple testing correction can then be applied to control the overall type I error. However, such an approach typically captures only a small proportion of the contributing genetic variants. One likely reason is that common and complex diseases result from the joint effects of multiple loci and environmental factors, each of which has a small individual contribution [1,2]. A variety of tests have been proposed to establish the joint association of multiple SNPs with the phenotype.

For such joint association analyses, the first category of statistical methods are those that use single SNP p-values or test statistics to construct a new joint test statistic. The Most Significant SNP method (MSS) uses the smallest p-value to declare significance. Fisher's method combines p-values by using negative of the twice of logarithm of product of p-values. Another group of test statistics pools SNPs with relatively strong signals from univariate tests, which include the sum of the *K *largest test statistics [3], the product of all the tests declared to be significant with some level *α *[4], the product of the *K *most significant p-values (Ranked Truncated Product; RTP) [5], and the weighted and truncated sum of logarithm of p-values [6].

Another category consists of strategies that modify the standard multivariate test statistics in order to reduce the effective degrees of freedom and hence improve the power [7-9]. Evaluation of some of the methods is presented in [10]. Some other methods use genetic similarity between individuals to establish multi-marker association in a genomic region with the phenotypes [2,11,12]. A comparison of the methods using the similarity measures and some additional references on multi-marker association tests can be found in [12].

The above multi-marker association tests are useful to establish the joint association of a set of SNPs with the trait. But brute-force searches to identify the subsets of associated SNPs contained on high-density SNP arrays are inefficient. Gene annotation databases group functionally relevant genes into biological pathways. Some of these pathways are likely to be involved in the etiology of complex diseases [13] and hence testing a few hundred such pathways to identify the subsets of genes involved in the diseases avoids a huge multiple testing burden. Thus pathway-based analyses can offer an attractive alternative to improve the power of GWAS, and may help us to identify relevant subsets of genes in meaningful biological pathways underlying complex diseases.

In the era of post-GWAS analysis there is considerable interest in pathway analysis, and several approaches for testing associations with pathways have been proposed. The Kolmogorov-Smirnov procedure is used to detect pathways containing a relatively high proportion of significant SNPs in [14] or significant genes [15]. The SNP Ratio Test (SRT) assesses association by comparing the proportion of significant SNPs within a pathway with those ratios in permuted data sets [16]. The Prioritizing Risk Pathways (PRP) method defines a risk score to identify risk pathways by integrating genetic factors and biological networks [17]. Combinations of univariate test statistics or univariate p-values for pathway association analysis is also considered in the literature. Using the Adaptive Ranked Truncated Product (ARTP) method [18] for gene-level p-values, a pathway association method has been proposed [19]. The sum of the Armitage trend test statistics of all of the SNPs within the pathway is used in [20]. Accounting for the correlation among the SNPs within the pathway, three approaches for combining univariate test statistics have been recently proposed [21]. Important factors for considerations in pathway analysis and a review of statistical methods for testing pathway associations are given in [22]; some interesting insights into the pathway analysis using the existing data bases are summarized in [23].

Most of the pathway association methods consider all the genetic variants (e.g. SNPs) within a pathway as possible risk factors [17,20,21]. If only a subset of genetic variants within a pathway has contribution to the disease then these methods may result in loss of power. To address this problem we propose a pathway-based analysis using a model selection criterion to identify a subset of associated genes within the pathway. In the proposed method, each gene is scored by the first principal component (FPC) of SNP genotypes, which effectively reduces the number of genetic variants considered for a model and hence reduces the degrees of freedom for joint analysis. On the basis of FPC scores, the gene subset is selected using LASSO penalized regression [24] combined with some model selection criterion. The p-value of the joint test is calculated by permutation of disease status among affected and unaffected individuals. For the GWAS pathway analysis, the False Discovery Rate (FDR) and the Family-Wise Error Rate (FWER) are applied to adjust for multiple testing.

## Methods

### The joint analysis using reduced gene subset

**Figure 1:**
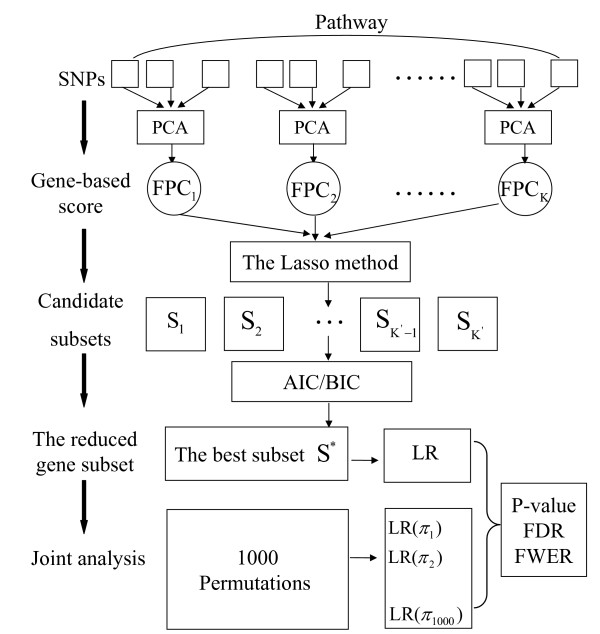
**The flowchart of the various steps of our method**.

Consider a pathway G with *K *genes g_1_,..., g_*K*_. Let **Y **denote the column vector of disease status for *n *individuals; the matrix X = (X_1_,..., X_*K*_) denotes genotype measurements on *n *independent individuals, where X_*j *_is the *n *by *p*_*j *_matrix of genotypes for *p*_*j *_SNPs within gene g_*j*_. The genotype measurements are coded as 0, 1 and 2, which correspond to the homozygous genotype for the major allele, the heterozygous genotype and the homozygous for minor allele respectively. In the following, various steps of the proposed method are described and the flowchart of the joint analysis is presented in Figure 1.

#### Gene-based score: the first principal component (FPC)

The first step is to calculate the gene-based scores for each of the *K *genes. It is well known that Principal Components Analysis (PCA) is an effective way to reduce dimensionality, and the First Principal Component (FPC) explains the maximum variance among all linear combinations [25]. In addition, the power of univariate tests using the FPC is comparable to the power of multivariate tests using the first few principal components [26]. Thus, it is feasible to consider the matrix of FPC of SNP genotypes within each gene as the gene-based score. The gene-based score effectively reduces the number of possible factors from $P=\sum_{j=1}^{K}p_{j}$ to *K *for pathway G, where *P *and *K *respectively denote the total number of SNPs and the total number of genes in G. When calculating FPCs, we choose to first center genotypes of each SNP over *n *individuals by subtracting the mean value. When the data set contains considerable numbers of missing genotypes, the R package *pcaMethods *[27] can be used to calculate FPC scores.

#### The FPC-based test statistic using all genes

If all *K *genes are used in the joint analysis then the pathway G can be tested using the LR statistic of *K *genes, which is given by

$$\begin{array}{l} \text{T(G)}=\text{LR(G)}\\ \;=\frac{\text{sup\{}p(Y,\beta |{\text{FPC}}_{\text{1}}...{\text{FPC}}_{\text{K}}):\beta \in \Theta \}}{\text{sup\{}p(Y,\beta |{\text{FPC}}_{\text{1}}...{\text{FPC}}_{\text{K}}):\beta \in \Theta_{0}\}} \end{array}$$

where *p*(**Y, β **| FPC_1_,..., FOC_*K*_) is the density function of the phenotype given *K *FPC scores, the parameter space Θ = {*β*_0_, *β*_1_,..., *β*_*K*_} and Θ = {*β*_0_}. The p-value of the test statistic T(G) can be approximated by $\chi_{K}^{2}$. If only a subset out the *K *genes is truly associated with the disease then the multivariate test including all genes may result in a loss of power due to a large number of degrees of freedom (see simulation results). Hence, we propose to perform the multivariate test using a reduced gene subset.

#### The FPC-based test statistic using the reduced gene subset

We perform the gene subset selection by first using the LASSO method to generate a series of candidate subsets and then select the best subset. The LASSO penalized log-likelihood function is given by

$$\log\text{L}(\beta |\lambda )=\log\text{L}(\beta )-n\lambda \sum_{j=1}^{K}\left|\beta_{j}\right|,$$

where the vector **β **= (*β*_0_, *β*_1_,...,*β*_*K*_) denotes regression coefficients of *K *gene-based scores, and logL(**β**) is the log-likelihood function. When the tuning parameter *λ *is first set to be zero, the corresponding subset S_1 _contains all the *K *genes. The parameter *λ *is gradually increased from *λ*_1 _= 0 to *λ*_2_, which is the smallest value to delete at least one gene from S_1_. The subset produced by *λ*_2 _is denoted as S_2_. By increasing the values of the parameter *λ*, the LASSO method produces a series of non-empty subsets {S_1_, S_2_,..., S_*K'*_}. In general, there is a nesting among the subsets, although they are not strictly monotonic. From these subsets, we select the best subset as the reduced gene subset to carry out the joint analysis. The standard approach to select the best subset is to use Cross-Validation (CV) or to optimize the quantity of the model selection criterion score such as the Akaike's Information Criterion (AIC) or the Bayesian Information Criterion (BIC). In the context of GWAS, the computation burden of CV is large as compared with AIC and BIC. Hence, we choose AIC or BIC as the gene subset selection criterion in our method. For subset S_j _(*j *= 1,..., *K*^'^), the AIC score can be expressed by

$$\text{AIC }({\text{S}}_{\text{j}})=-2\log\text{L}(\hat{\beta }({\text{S}}_{\text{j}}))+2\text{v}({\text{S}}_{\text{j}}),$$

where $\log\text{L}(\hat{\beta }({\text{S}}_{\text{j}}))$ is the maximized log-likelihood function of S_j_, and v(S_j_) denotes the number of genes contained in S_j_. The BIC score of S_j _is given by

$$\text{BIC }({\text{S}}_{\text{j}})=-2\log\text{L}(\hat{\beta }({\text{S}}_{\text{j}}))+\log\text{(}n)\text{v}({\text{S}}_{\text{j}}),$$

where *n *is the sample size. Since the penalty function in BIC, log(*n*)v(S_j_), is usually larger than the one in AIC, 2*ν*(S_j_), the subset selected by BIC are usually contained within the subset selected by AIC. Denote the reduced subset selected by AIC or BIC by S* = {g_(1)_,..., g_(__*m*__)_}. The test statistic of pathway G using S* is defined as the LR test statistic of S*

$$\begin{array}{l} \text{T(G)}={\text{LR(S}}^{\text{*}})\\ \;=\frac{\sup\{p(Y,\beta |{\text{FPC}}_{\left(1\right)}...{\text{FPC}}_{\left(m\right)}):\beta \in \Theta \}}{\sup\{p(Y,\beta |{\text{FPC}}_{\left(1\right)}...{\text{FPC}}_{\left(m\right)}):\beta \in \Theta_{0}\}}, \end{array}$$

where Θ = {*β*_0_, *β*_(1)_,..,*β*_(__*m*__)_}, Θ_0 _= {*β*_0_}, and FPC_(1)_,..., FPC_(__*m*__) _denote the FPC scores of genes *g*_(1)_,..., *g*_(__*m*__)_.

#### P-value, False Discovery Rate (FDR) and Family-Wise Error Rate (FWER)

Although the nominal distribution of T(G) can be approximated by $\chi_{m}^{2}$, the p-value of pathway G can no longer be obtained directly from the chi-square distribution. Therefore the p-value for pathway G is obtained using a permutation procedure that is based on the permutation of disease status among affected and unaffected individuals. Note that the purpose here is not to test the association of the fixed set subset S*, but to test the association of pathway G using this reduced gene subset. Hence we need to select the reduced subset in each permuted data set for computing the p-value of pathway G. In each permutation *π*, LASSO is applied to generate a series of candidate subsets using the same procedure. Under the null hypothesis, the test statistic in the permuted data set should have the same nominal distribution as the one in the original data ($\chi_{m}^{2}$). Hence, the size of the selected subsets in permutation *π *is also restricted to *m *(see the Discussion section for additional discussion). Denote the gene subset selected by LASSO in the permutation *π *as S*(*π*). The test statistic of pathway G in *π *is given by

$$\begin{array}{l} \text{T(G(}\pi \text{))}={\text{LR(S}}^{\text{*}}(\pi ))=\\ \frac{\sup\{p(Y(\pi ),\beta |{\text{FPC}}_{\left(1\right)}(\pi )...{\text{FPC}}_{\left(m\right)}(\pi )):\beta \in \Theta \}}{\sup\{p(Y(\pi ),\beta |{\text{FPC}}_{\left(1\right)}(\pi )...{\text{FPC}}_{\left(m\right)}(\pi )):\beta \in \Theta_{0}\}} \end{array}$$

where **Y **(*π*) denotes disease status in permutation *π*, and FPC_(1)_(*π*),..., FPC_(m) _(*π*) denote the FPC scores of the *m *genes in S* (*π*). The p-value of pathway G is defined as

$$\begin{array}{l} \text{p-value(G})=\\ \frac{\#\text{ of permutations }\pi \text{ with T(G(}\pi \text{))}>\text{T(G)}}{\text{\# of permutations }\pi }. \end{array}$$

For GWAS, FDR and FWER are used to adjust for the multiple hypothesis testing. To be able to compare across several pathways, we first calculate the normalized LR test statistics denoted here as NLR(G) and NLR(G(*π*)). The NLR(G) is written as

$$\text{NLR(G)}=\frac{\text{T(G)}-\text{mean(T(G(}\pi \text{)))}}{\text{SD(T(G(}\pi \text{)))}}.$$

The FDR of pathway G is defined as

$$\begin{array}{l} \text{FDR(G)}=\\ \frac{\text{\# of permutations with NLR(AG,}\pi \text{)}\geq \text{NLR(G)}}{\text{\# of all obeseved AG with NLR(AG)}\geq \text{NLR(G)}} \end{array}$$

where AG denotes any pathway in GWAS. The FWER is defined as

$$\begin{array}{l} \text{FWER(G)}=\\ \frac{\text{\# of permutaions }\underset{\text{AG}}{\max}\{\text{NLR(AG,}\pi \text{)\}}\geq \text{NLR(G)}}{\text{\# of permutations }}. \end{array}$$

### Simulation description

An extensive simulation study is conducted to compare the proposed joint analysis with the methods MSS, RTP, the Admixture Maximization Likelihood (AML) [8] method. To mimic a GWAS (more details in RESULTS), we simulate genotypes for SNPs within associated pathways using a published GWAS data set. To model the disease status based on multiple variants, the number of associated genetic variants and the estimates for relative risks are all obtained from this data set. In each simulated data set, we sample 1350 affected and 1350 unaffected individuals, which is similar to the sample size of the GWAS study. In both the simulated and original GWAS data sets, we map the SNPs to a gene if they are within 5 kb of the gene. For each set of parameters, 500 replicates are simulated. The power and type I error are derived as the proportion of replicates with p-values ≤0.05.

#### Simulated Pathways

To reflect the power of different methods to detect small, medium and large pathways, we choose three different pathway patterns in the simulations. The three pathways considered are "TNFR2 Signaling Pathway", "Fructose and mannose metabolism" and "Cytokine-cytokine receptor interaction"; called here as pathway patterns 1, 2 and 3 respectively. The pathways and the genes within them were constructed using two existing databases (more details are given in the RESULTS section). The three pathways capture a wide spectrum of the constituent gene numbers and effect sizes (Table 1) observed in GWAS pathway analysis.

**Table 1:** The information of the three pathway patterns used in simulation

*Pathway*	*# of total genes (SNPs)*	*# of assoc genes*	*# of causalSNPs*	*Minor Allele Frequency (MAF)*	*Estimate Relative Risk (ERR)*
TNFR2 Signaling Pathway (pattern 1)	15 (159)	2	4	0.340, 0.416, 0.298, 0.317	1.070, 1.081, 1.117, 1.130
Fructose and mannose metabolism (pattern 2)	35 (339)	4	6	0.163, 0.353, 0.359, 0.359, 0.424, 0.332	1.113, 1.077, 1.063, 1.101, 1.105, 1.088
Cytokine-cytokine receptor interaction (pattern 3)	208 (1957)	19	39	0.409, 0.491, 0.491, 0.275, 0.397, 0.315, 0.210, 0.073, 0.372, 0.415, 0.281, 0.219, 0.472, 0.305, 0.355, 0.435, 0.112, 0.323, 0.373, 0.044, 0.063, 0.227, 0.430, 0.070, 0.161, 0.355, 0.161, 0.034, 0.156, 0.417, 0.420, 0.169, 0.210, 0.175, 0.183, 0.269, 0.194, 0.177, 0.499	1.100, 1.065, 1.064, 1.090, 1.055, 1.086, 1.167, 1.172, 1.057, 1.084, 1.141, 1.081, 1.095, 1.149, 1.072, 1.092, 1.087, 1.062, 1.132, 1.134, 1.162, 1.094, 1.060, 1.247, 1.078, 1.062, 1.096, 1.249, 1.123, 1.070, 1.089, 1.073, 1.087, 1.093, 1.074, 1.064, 1.087, 1.123, 1.071

#### Genetic variants associated with disease

We simulate both associated and non-associated genetic variants within the pathway with the assumption that not all the genetic variants in the pathway are disease associated. Associated genes and SNPs are completely determined by gene-based and SNP p-values in GWAS analysis. First, based on the LR test with FPC, a list of associated genes (p-value ≤ 0.05) within the pathway is generated. For an associated gene, all the SNPs with p-values ≤ 0.05 are divided into several groups on the basis of the criterion that the SNPs within the same group are in strong Linkage Disequilibrium (LD), i.e. *r*^2 ^≥ 0.8. In each group, we select the one with the smallest p-value as the causal SNP. The Minor Allele Frequencies (MAFs) and Estimated Relative Risks (ERRs) of causal alleles are obtained from GWAS study (displayed in Table 1). In each underlying pathway pattern, the relative risks (RRs) of risk alleles are first equal to ERRs. To evaluate the performance of the association statistic under a spectrum of RRs, we then multiply each of the ERRs by a factor less than unity.

#### Simulated genotypes and disease status

To retain the observed LD structure within the genes, the haplotype frequencies of the control samples in the GWAS are estimated using *PLINK *[28]. For simulation, we generate a sample of haplotypes with probabilities proportional to the estimated haplotype frequencies. Under the assumption of Hardy-Weinberg Equilibrium (HWE), two haplotypes are selected at random to form a pair of unphased haplotypes for an individual. Finally, the genotypes for SNPs are obtained by taking the corresponding alleles from the haplotype pairs. Given the RRs and MAFs, the disease status of an individual is inferred using a multiplicative relative risk model [12]. Using this procedure we have generated 500 replicate data sets of 1350 cases and 1350 controls.

### Methods for comparison

Let us denote our pathway association tests based on FPCs with gene subset selection using AIC and BIC criteria as FPC_AIC and FPC_BIC respectively; the method that uses all the genes for the pathway association without the model selection is denoted as FPC_FULL. The three methods MSS, RTP and AML have been shown to perform well for detecting the association in comparison with some other existing methods [8]. To illustrate the benefit of the gene subset selection, we compare the methods FPC_AIC and FPC_BIC with FPC_FULL. The modified GSEA algorithm [15] is alternative method to carry out the pathway analysis in GWAS. However, this method cannot be applied in a candidate pathway analysis since it needs gene p-values on the whole genome. Hence, we do not include this method for comparison with other methods in simulation studies.

The test statistic of the MSS method is the smallest p-value of single SNP tests of all the SNPs in a pathway. The test statistic of the RTP method [5] is the product of the smallest *k *p-values. In our simulation, we choose *k *= 5 (RTP_5) and *k *= 10 (RTP_10). The AML method supposes that SNPs detectable in the experiment have the same contribution to the disease and all genotyped SNPs are independent. Under these assumptions, the EM algorithm simultaneously estimates the proportion of associated SNPs *α *and their average effect *ζ*. The LR statistic is written as

$$\begin{array}{l} \lambda (\alpha ,\zeta )=\sum_{j=1}^{P}\log((1-\alpha )+\alpha (-\text{exp}(-z_{j}^{2}/2)\\ +\exp(-{\left(z_{j}-\zeta \right)}^{2}/2)/2+\exp(-{\left(z_{j}-\zeta \right)}^{2}/2)/2), \end{array}$$

where *P *is the total number of SNPs, *z*_*j *_denotes the square root of *χ*^2 ^statistic at locus *j*. In MSS, RTP and AML methods, the single SNP p-values are based on the Cochan-Armitage trend test.

## Results

In this section we report the results in parts. The first part is a comparison of power for our proposed method relative to four other methods. In the second part, we evaluate the performance of gene subset selection and compare it with the gene selection approach based on corrected p-values. In the third part, our method is applied to the psoriasis GWAS data from the Collaborative Association Study of Psoriasis (CASP) [29] to detect susceptibility pathways. This study is part of the Genetic Association Information Network (GAIN) consortium. The data set consists of 438 K SNPs genotyped on 1409 psoriasis cases and 1436 controls. As part of our ongoing meta-analysis study, we have imputed additional SNPs on this data set. After the standard quality control filters for GWAS (SNP call rate and sample call rate ≥90%, Minor Allele Frequency (MAF) >0.005 and Hardy-Weinberg equilibrium (HWE) p-value> 10^-7^, relationship testing and outlier detections), we report our analysis based on 529,651 SNPs in 1349 cases and 1372 controls.

### Power of joint analysis

**Figure 2:**
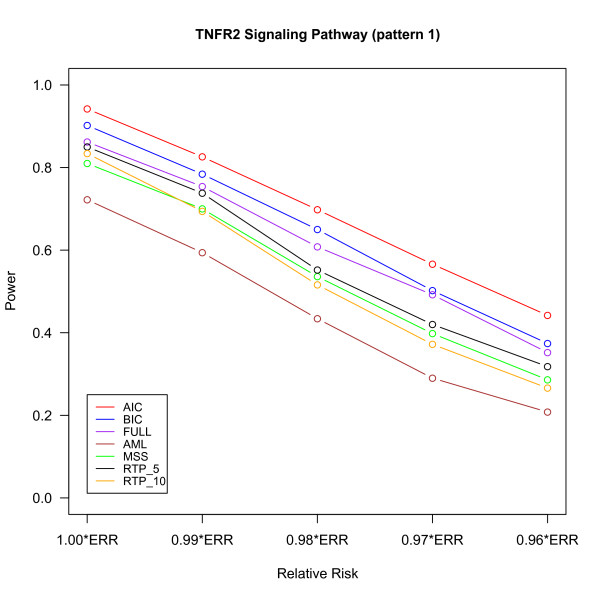
**Power versus relative risk in "TNFR2 Signaling Pathway" (pattern 1)**. The power of several methods is shown for different relative risks. ERR denotes the estimated relative risk from the real data set. The power are based on 500 replicates and defined as the proportion of replicates declared significant at the significant level 0.05.

**Figure 3:**
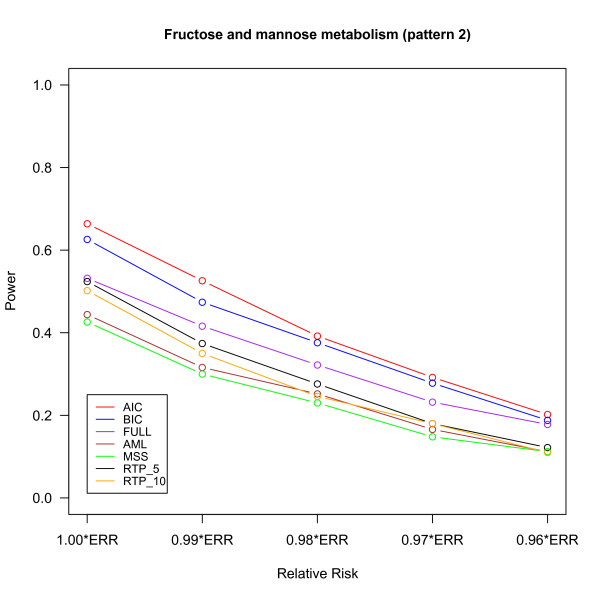
**Power versus relative risk in "Fructose and mannose metabolism" (pattern 2)**.

**Figure 4:**
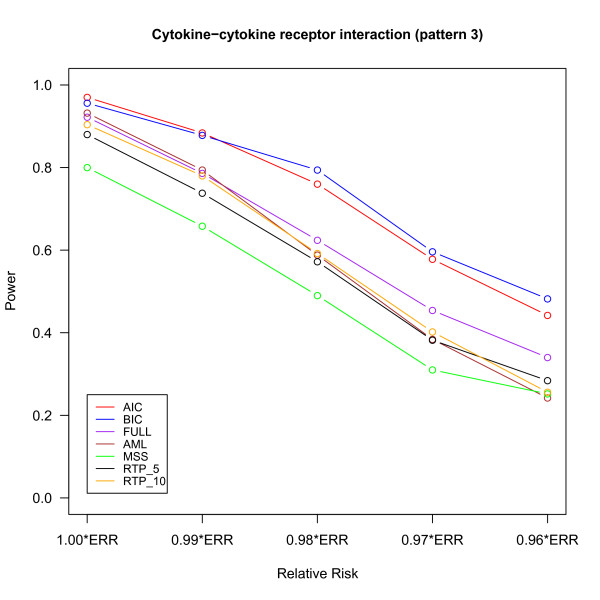
**Power versus relative risk in "Cytokine-cytokine receptor interaction" (pattern 3)**.

The power of different methods under the three pathway patterns are shown in Figures 2, 3 and 4. The details of the power, the 95% confidence intervals of estimated probabilities and the p-value of McNemar's test of difference in powers between FPC_BIC and other methods are given in Additional file 1, Tables S1, S2 and S3. For the pathway patterns 1 and 2 (Figures 2 and 3), the FPC_AIC is slightly more powerful than FPC_BIC; but it is less powerful than FPC_BIC for the pathway pattern 3 (Figure 4). The possible reason is that FPC_AIC tends to select too many genes when the percentage of non-associated genes is large.

FPC_AIC or FPC_ BIC outperformed other methods under all the 3 pathway patterns. The power of FPC_FULL is only slightly less than FPC_BIC in pathway pattern 1. As the number of non-associated genes increases in the pathway patterns 2 and 3, the power of FPC_FULL decreases and it is less than FPC_AIC or FPC_BIC due to large degrees of freedom associated with this statistics. One advantage of FPC_FULL is fast computation, because its p-value can be approximated by a chi-square distribution. FPC_AIC or FPC_BIC performs much better than the AML and MSS methods. The AML method assumes that all SNPs are independent and all causal SNPs have the same effects. Under these two assumptions AML has been shown to have a good performance in [8]. However, if some SNPs are highly correlated with truly associated SNPs, then AML would overestimate the number of causal SNPs [8] and may also reduce the effects of truly associated SNPs. MSS may result in loss of power if there are many associated SNPs because it uses only the most significant SNP to declare significance. Although RTP had higher power than MSS and AML, its power was still lower than FPC_AIC or FPC_BIC. The performance of the RTP method depends on the number of associated genes in the pathway (Figures 2, 3 and 4) and hence pre-specification of number of SNPs to for this method is important [8].

We evaluated the performance of the methods under a spectrum of RRs by multiplying each of the ERRs by a factor from 1.00 to 0.96. For example, under multiplicative model, the combined RR (1.00*ERRs) for pattern 1 with 4 causal SNPs is 1.46(1.07×1.081×1.117×1.13), but when the factor 0.99 is multiplied to ERR (0.96*ERRs), the combined RR drops to 1.24 (1.46×0.96^4^) and results in reduction of powers for all the pathway association tests due to the factors like LD pattern of the genes, effect sizes and MAF of the causal SNPs. Note that the powers for all the methods are generally low for 0.96*ERRs, but the performance of FPC_AIC or FPC_BIC was still the best. Table 2 summarizes the type I error, 95% confidence interval for estimated probability and the p-value of McNemar's test to compare the difference in type I errors between FPC_BIC and other methods. All the methods had a type I error controlled around 0.05 under all settings.

**Table 2:** Type I error of different methods

* Methods*	TNFR2 Signaling Pathway (pattern 1)	Fructose and mannose metabolism (pattern 2)	Cytokine-cytokine receptor interaction (pattern 3)
	
	Type I error (95% CI) P-value	Type I error (95% CI) P-value	Type I error (95% CI) P-value
FPC_AIC	0.060 (0.039,0.081) 0.302	0.058 (0.038,0.078) 0.326	0.054 (0.034,0.074) 0.653
FPC_BIC	0.050 (0.031,0.069) NA	0.048 (0.029,0.067) NA	0.048 (0.029,0.067) NA
FPC_FULL	0.058 (0.038,0.078) 0.492	0.054 (0.034,0.074) 0.673	0.048 (0.029,0.067) 0.932
AML	0.062 (0.041,0.083) 0.346	0.042 (0.024,0.060) 0.653	0.034 (0.018,0.050) 0.285
MSS	0.058 (0.038,0.078) 0.523	0.050 (0.031,0.069) 0.933	0.042 (0.024,0.060) 0.689
RTP_5	0.058 (0.038,0.078) 0.536	0.046 (0.028,0.064) 0.917	0.042 (0.024,0.060) 0.673
RTP_10	0.064 (0.043,0.085) 0.258	0.048 (0.029,0.067) 0.922	0.044 (0.026,0.062) 0.797

For each of the methods, we presented the type I error, the 95% confidence interval of all estimated probabilities, the p-value of McNemar's test for the difference of type I error between FPC_BIC and other methods. The type I error was calculated on the basis of 500 replicates at significance level 0.05.

### Evaluation of gene subset identification

It is important to evaluate the performance of gene subset identification using our approach to understand the proportion of true and false positive genes in the selected subset of genes. Apart from the AIC and BIC discussed above, we also consider the classical approach of identifying the gene subset by using the multiple test correction. Under multiple testing (denoted as gene-test here), the p-value for each gene is obtained using the gene-score LR test and then it is corrected for the total number of genes tested under the Bonferroni method. The subsets of associated genes using this procedure are those that had significant corrected p-values (<0.05). The performance of the methods is examined by two measurements: Positive Selection Rate (PSR) to describe the ability of the method to select the associated genes and False Discovery Rate (FDR) for the purity of the selection. PSR is defined as

$$\text{PSR}=\frac{\#\;\;\text{genes truly declared associated}\;}{\#\;\text{ associated genes}};$$

FDR is defined as

$$\text{FDR}=\frac{\#\;\text{ genes falsely declared associated}\;}{\#\;\text{ genes declared associated}}.$$

The FDR of a pathway defined earlier is slightly different from the FDR defined here. The former is described for real data analysis in which the truly associated genes are unknown whereas the above definition of FDR is for the simulation study in which all the associated genes are known.

Table 3 summarizes the average PSR and FDR over 500 replicates under the three pathway patterns with the ERR for FPC_AIC, FPC_BIC and the gene-level association test (gene-test). Compared with BIC, AIC tended to select the gene subsets with more genes due to its small penalty function and hence results in high FDR. For example, for the pathway pattern 1, the PSR of FPC_AIC was 0.88, which meant that almost all associated genes were selected by the AIC criterion but with high FDR. Although the PSR of BIC was lower than AIC, BIC enjoyed a much lower FDR. In the pathway pattern 1, BIC has controlled the FDRs around 0.05, which ensures that majority of genes declared associated by BIC are likely to be associated. For pathway pattern 3, the average number of genes selected by AIC was found to be 38.95 of which 10.17 were truly associated and 28.78 were false positives. Compared to the total number of genes in this pathway (208), AIC not only dramatically reduced the number of candidate genes to a reasonable number, but also ensured that a large proportion of associated genes (53.5%) were selected. This result suggests that AIC provides a possible candidate gene subsets for further investigation in follow-up studies. The average number of selected genes by BIC was 4.01, among which 3.65 genes were truly associated and 0.36 genes were falsely associated. Thus genes selected by BIC could be considered as important genes as the FDR associated with this approach is very small. It should be noted that the type I error of AIC is kept around 0.05 in pathway detection, although AIC is too liberal in gene identification.

**Table 3:** PSR and FDR for Gene subset identification of different methods

*Methods*	*TNFR2 Signaling Pathway (pattern 1)*		*Fructose and mannose metabolism (pattern 2)*		*Cytokine-cytokine receptor interaction (pattern 3)*	
			
	PSR	FDR	PSR	FDR	PSR	FDR
FPC_AIC	0.880	0.524	0.473	0.665	0.535	0.739
FPC_BIC	0.318	0.031	0.243	0.093	0.192	0.090
Gene-test	0.310	0.024	0.185	0.075	0.085	0.025

The PSR and FDR of the gene-test were much lower than those of AIC under all the pathway patterns. In the pathway pattern 1, there was little difference between the PSR of the gene-test and that of BIC. But, the PSR of gene-test is lower than that of FPC_BIC in the other two pathway patterns. It is likely that the Bonfferoni correction becomes too conservative as the number of genes within pathway increases. The FDR of BIC is higher than that of gene-test, but is still controlled at a reasonable level (<0.10). These results showed that our method is more powerful than the gene-test for gene identification and has a comparable FDR when using BIC as the selection criterion.

### Application to a published psoriasis GWAS data set

Psoriasis is a chronic inflammatory skin disease that is marked by a complex interplay of Dendritic Cells (DCs), T-cells, cytokines, and downstream transcription factors as part of a self-sustaining type 1 cytokine network [30]. Here, we applied the proposed method in a published psoriasis GWAS data set from the CASP to identify susceptibility pathways and important genes. We mapped all 529,651 SNPs to genes within the 5 kb upstream and downstream region. If the SNPs are mapped to multiple genes using this definition, we used a hierarchical mapping scheme (coding> intronic> 5'utr> 3'utr) following [31]. The information on gene ID, gene names, and their start and end positions on a chromosome was downloaded from the National Center for Biotechnology Information (NCBI)'s Genome database http://www.ncbi.nlm.nih.gov/Genomes/. The quantile-quantile plot in the original study [29] showed that signals in MHC are much stronger than those in non-MHC. Hence, our analysis only considered non-MHC genes across the whole genome to aviod the bias arising from strong signals in the MHC. We generated 201 annotated pathways from KEGG Pathway Database and 320 annotated pathways from Biocarta Pathway Database by using Database for Annotation, Visualization and Integrated Discovery (DAVID) [32]. We only examined the 247 pathways with at least 15 genes to avoid testing overly narrow pathways.

We applied the proposed methods FPC_AIC, FPC_BIC, GSEA algorithm, and the AML to carry out the pathway-based analysis. Table 4 summarizes the pathways detected by at least one method. The proposed method with the BIC criterion detected 6 biologically plausible pathways for psoriasis (1 to 6). The detected pathways under the AIC criteria were similar to the ones under the BIC criteria (1-3, 5-7) and these are also reported in previous studies [33-38]. Although "NF-kB Signaling Pathway" reported in the original paper [29] was not declared to be associated due to slightly high FWERs (AIC: 0.144, BIC: 0.078), it had p-values less than 0.001 and FDRs less than 0.05. The methods FPC_AIC or FPC_BIC also identified important genes within the detected pathways (Table 5). By comparing the total number of genes (set size) with the number of genes identified by the proposed approach (subset size), it can be seen that our approach effectively identified a few associated genes within the pathway. At the same time, our analysis is able to identify nearly all of the genes reported by previous studies, such as IL13, STAT2, IL23R, IL12B, IL23A, IL4, TNFAIP3 [29], and RAD50 [39]. The findings demonstrate that the proposed method is a promising way to identify associated pathway and important genes in GWAS.

**Table 4:** The pathways detected by at least one method in GAIN data set

	*FPC-BIC*	*FPC-AIC*	*GSEA*	*AML*
*Pathway*	*P-value (FDR, FWER)*	*P-value (FDR, FWER)*	*P-value (FDR, FWER)*	*P-value (FDR, FWER)*
1. Cytokine Network	<0.001 (0.006, 0.006)	<0.001 (0.001, 0.001)	0.020 (0.182, 0.945)	0.888 (0.911, 1.000)
2. Jak-STAT signaling pathway	<0.001 (0.004, 0.008)	<0.001 (0.001, 0.002)	0.001 (0.017, 0.080)	0.104 (0.518, 1.000)
3. Dendritic cells in regulating TH1 and TH2 Development	<0.001 (0.005, 0.015)	<0.001 (0.004, 0.019)	0.108 (0.448, 1.000)	0.634 (0.906, 1.000)
4. Cytokines and Inflammatory Response	<0.001 (0.005, 0.020)	0.001 (0.006, 0.057)	0.127 (0.455, 1.000)	0.710 (0.917, 1.000)
5. Cytokine-cytokine receptor interaction	<0.001 (0.005, 0.023)	<0.001 (0.004, 0.027)	0.004 (0.090, 0.534)	0.005 (0.494, 0.624)
6. Role of BRCA1, BRCA2 and ATR in Cancer Susceptibility	<0.001 (0.005, 0.037)	<0.001 (0.001, 0.001)	0.243 (0.560, 1.000)	0.014 (0.390, 0.871)
7. TNFR2 Signaling Pathway	0.001 (0.009, 0.099)	<0.001 0.004, 0.021	0.249 (0.559, 1.000)	0.303 (0.641, 1.000)
8. Calcium signaling pathway	0.022 (0.166, 1.000)	0.001 (0.040, 0.591)	<0.001 (0.009, 0.009)	0.002 (0.362, 0.655)
9. Axon guidance	0.017 (0.165, 0.997)	0.040 (0.139, 1.000)	0.001 (0.005, 0.010)	0.238 (0.600, 1.000)
10. ECM-receptor interaction	0.792 (0.858, 1.000)	0.392 (0.461, 1.000)	<0.001 (0.004, 0.012)	0.197 (0.600, 1.000)
11. Cell adhesion molecules (CAMs)	0.332 (0.520, 1.000)	0.011 (0.081, 0.957)	0.001 (0.007, 0.027)	0.023 (0.363, 0.994)

All the p-values, false discovery rates (FDRs) and family-wise error rates (FWERs) were obtained via 1000 permutations. Gene p-value in pathway enrichment method was the p-value of the most significant SNP within gene. The AML method was based on the test statistic of Cochan-Armitage trend test.

**Table 5:** The identified genes by AIC or BIC in their 5 common detected pathways

*Pathway*	*Set size*	*Subset size*	*Identified genes*
Cytokine Network	17	5/1	IL13, IL4, IL9, IL5, IL2
Jak-STAT signaling pathway	126	25/5	IL13, IL23R, IFNE1, STAT2, IL12B, IL4, IL9, IL29, IL5, IL6, IL5RA, IL2, PIK3R5, PIK3R2, EP300, PIK3CG, CSF2RB, TSLP, CNTFR, IFNA21, OSMR, IFNGR1, PIAS4, PIK3CB, SOCS4
Dendritic cells in regulating TH1 and TH2 Development	19	4/1	IL13, IL4, IL5, ANPEP
Cytokine-cytokine receptor interaction	208	49/6	IL13, IL23A, IL23R, IL1R2, IL12B, IFNE1, IL4, IFNA21, PF4V1, IL9, IL29, INHBA, IL5, FAS, OSMR, CCL22, FLT3, TNFRSF21, CSF1, CXCL11, CNTFR, IL1B, IL18RAP, TGFB2, CCL23, IL8RB, IL5RA, IL6, IL11RA, IL2, TNFRSF19, EGF, CCL3, IFNAR2, CCR6, CSF1R, TNFRSF8, PDGFRA, CSF2RB, TNFSF8, CCL1, TSLP, TNFRSF1A, KDR, IL17RB, IFNGR1, TNFRSF11B, IL22RA2, PDGFRB
Role of BRCA1, BRCA2 and ATR in Cancer Susceptibility	21	3/1	RAD50, BRCA2, RAD9A

Column "subset size" (# by AIC/# by BIC) presents the subset sizes selected by AIC and BIC, respectively. Column "selected genes" presents the identified genes by AIC and BIC, where genes with underline denote the ones selected by both AIC and BIC, and genes with no underline denote the ones selected only by AIC.

The modified GSEA algorithm identified 4 pathways (8-11). Although no common pathways were detected by both the FPC_AIC or FPC_BIC and the modified GSEA algorithm, the p-values of pathways 2, 5 and 8 were less than 0.005 for both of the two approaches. FPC_AIC and FPC_BIC were not able to identify pathways 10 and 11 partly due to the fact that no genes with significant p-values within these 2 pathways were found. The smallest gene p-values within pathways 10 and 11 are respectively 0.0197 and 0.004. In the modified GSEA algorithm, the enrichment score is constructed by using all of the genes within pathways, so the large percentage of non-associated genes will result in loss of power. Although the modified GSEA algorithm can highlight genes with extreme statistics of association by setting the parameter *p *> 1 in the enrichment score, the authors recommend using *p *= 1[15]. Otherwise, the modified GSEA algorithm suffers from the need to select the appropriate value for *p*. Compared to GSEA, the proposed approach with AIC or BIC would effectively increase the power to detect the association since the joint analysis is performed in a reduced gene subset. The AML method did not detect any pathway, but it also has p-values 0.005 and 0.002 for pathways 5 and 8. In our ongoing meta-analysis including multiple cohorts, we find that some of the pathways identified by our analysis in GAIN are also replicated in other cohorts. Complete Meta pathway results will be reported later.

## Discussion

Genes rather than SNPs are considered the basic units in our method, which is expected to reduce the degrees of freedom of the pathway test statistic. As part of our pathway association, we conducted gene-level association tests based on FPC scores using published psoriasis GWAS data and compared the result with single SNP analysis. Besides the genes reported by in the original paper [29], our gene-level analysis identified several additional genes (See details in Additional file 2, Table S4). It is likely that, if a gene contains a number of SNPs with medium-size effects, the gene-based score combines their information and hence increases the effect size. Hence, our proposed method using gene-based scores should increase the power to detect an association.

In the analysis of empirical data, the proposed method identified a number of biologically plausible pathways for psoriasis. The pathogenesis of psoriasis is characterized by skewed cytokine levels of pro-inflammatory and anti-inflammatory cytokines [34]. Cytokines are the hormonal messengers responsible for most of the biological effects in the immune system. Cytokines can be functionally divided into two groups: those that are pro-inflammatory and those that are essentially anti-inflammatory [38]. A subgroup of the T lymphocytes, also known as helper T cells, is regarded as being the most prolific cytokine producers. This subset can be further subdivided into Th1 and Th2, and the cytokines they produce are known as Th1-type cytokines and Th2-type cytokines. Th1-type cytokines (IL-1, IL-2, IFN-*γ *and TNF-*α*) tend to produce the pro-inflammatory responses, while Th2-type cytokines (IL-4 and IL-10) have an anti-inflammatory response. Thus, pathways "Cytokine Network" and "Cytokine - cytokine receptor interaction" are relevant to the etiology of psoriasis. Our results highlighted a possible role for dendritic cells in regulating TH1 and TH2 development. DCs are key sentinels of the immune system, bridging the gap between innate and adaptive immunity [36]. A facet of DCs is that these cells may have alternative differentiation pathways that stimulate differing T-cell subsets that typify Th1 vs. Th2 skin diseases [37]. Collectively, cytokines like TNF-*α *and IFN-*γ *induce a wide variety of responses including: STAT1 stimulation followed by expression of downstream response genes and NF-*κ *B signaling pathways seen in psoriatic lesions [30]. NF-κB activation regulates the transcription of other pro-inflammatory genes [35]. Evidences suggest that BRCA1 part of the "Role of BRCA1, BRCA2 and ATR in Cancer Susceptibility" pathway significantly enhances the ability of TNF-*α *or IFN-*γ *to activate transcription from the promoters of NF-κB target genes. Together, this information suggests that BRCA1 may play a role in cell life-death decisions following cell stress by modulation of the activity of NF-κB [33]. Thus, "Jak-STAT signaling pathway", "TNFR2 signaling Pathway" and "Role of BRCA1, BRCA2 and ATR in Cancer Susceptibility" are involved in the regulation of cellular responses to cytokines with the aim to providing an insight to the molecular mechanism involved in psoriasis.

In our analysis we compared results from the proposed method to that of the GSEA method, since both of the two methods consider genes as basic genetic variants. We also note that there are other existing pathway-based methods for GWAS [14,16-21]. However, most of these methods are taking SNPs as basic genetic variants in the analysis, so we did not choose them as methods for comparison. Our annotation of SNPs to genes is based on 5 kb window size around the gene. There are several options for annotation of SNPs to gene such as 10 kb, 50 kb, and even 500 kb [40,41,15] window sizes around the gene but we used only 5 kb window size because a large window size would result in too many overlapping genes. The first principal component of SNP genotypes is used to capture the information in a gene, which is reasonable but not necessary. Many alternative ways are feasible, such as the most significant SNP test statistic used in the GSEA method [15], the first few principal components discussed in [26].

We have also performed several diagnostic tests to examine the proposed pathway approach. First to find out whether our defined gene-based score is affected by the length of the associated genes, we performed Welch's t-test to compare the mean numbers of SNPs in the "associated" genes (p-value ≤ 0.05) vs those in "non-associated" genes (p-value > 0.05). The mean numbers of SNPs in the "associated" and "non-associated" genes are 15.18 and 16.07, respectively; combined estimate of standard deviation of two samples is 0.82; the p-value for the test of equality of the mean gene lengths is 0.276; 95% confidence interval for the mean difference is (-2.79, 0.71). This result shows that gene-based test using FPC is not biased due to the number of SNPs in the genes. Second, we have only used the first principal component of SNP genotypes to capture the information in a gene, which is may not account for all the variation within the gene. To assess whether the FPC captures all the information within the genes, we performed an alternative gene-level analysis by using the first two principal components. The LR test was done for each of the two principal and the smallest p-value as is taken as the gene-level p-value. We compared the top 50 genes in these two gene-level analyses and found that the FPC did not detect only 5 genes detected by the first two principal components. This findings show that FPC contains most of the information across multiple SNPs within the same gene. Hence, it is feasible to consider the FPC as the gene-based score to represent genes without increasing the multiple test burdens with many principal components [26].

In the proposed permutation procedure, the size of the gene subset is kept to be the same as the one in the original analysis (*m*). This guarantees the LR test statistics of the original data and permuted data to have the same nominal distribution ($\chi_{m}^{2}$) under the null hypothesis. If the size of gene subset is re-computed in permutation *π *(denoted by *m*(*π*)), the corresponding LR test statistic approximately follows $\chi_{m(\pi )}^{2}$ under the null hypothesis. When *m*(*π*) is not equal to *m*, the test statistics in original data and in the permuted data will not follow the same nominal distribution and hence it is difficult to compare both of them. As discussed in [42], one disadvantage of the permutation procedure is that the test statistic may not follow the overall mean and standard deviation. To overcome this problem a restandardization procedure was proposed by combining the randomization with the permutation procedure [42]. Currently, we are extending this idea to work on a novel procedure for the calculation of p-values. However, it is beyond the scope of this article.

The method proposed here can be extended in several ways. For example, we have considered only the disease trait (affected vs. unaffected) but this can easily be extended to quantitative traits as well. This approach can also be used to perform whole genome prioritized analysis such as the SNPs in the transcription factor binding sites. Association through Copy Number Variants (CNVs) and meta-analysis can also performed under this framework. To relieve the multiple testing burdens, our pathway analysis uses available knowledge on annotated biological pathways. As a result, it is important that these annotated pathways are well supported by data to avoid false positive findings.

## Conclusions

In this paper, we proposed a pathway-based approach to examine the joint effects of a biological pathway through jointly testing the reduced gene subset. Apart from identifying susceptibility pathways, our method also provides a solution to detect important genes using gene selection criteria. The simulation results show that our method effectively increased the power to detect the associated pathways compared to a number of widely used approaches. In the GWAS of psoriasis in GAIN population, our method using the AIC criterion identified 6 biologically plausible pathways and several important genes for psoriasis. The findings demonstrate that our proposed method is a promising way to discover susceptibility pathways and identify important genes in GWAS.

## Authors' contributions

Conceived and designed the experiments: AT and JZ. Performed the gene annotation: SG. Analyzed the data: JZ, SG, MS and JL. Wrote the paper: JZ. Made the major edits: AT, SG, JL and MS. All of the authors read and approved the manuscript.

## Supplementary Material

Additional file 1 **Additional Tables S1, S2, S3 listing the power of different methods**. Tables S1, S2 and S3 respectively summarizes the powers of different methods for three pathways "TNFR2 Signaling Pathway", "Fructose and mannose metabolism" and Cytokine-cytokine receptor interaction" in simulation studies. Click here for file

Additional file 2 **Additional Table S4 listing the top 10 associated genes in GAIN data set**. Table S4 lists the top 10 genes associated in psoriasis GAIN data set by using the FPC-based gene level test. Click here for file
