# Supplementary material for: Pathway-based analysis using reduced gene subsets in genome-wide association studies
Source: BMC Bioinformatics. 2011 Jan 12;12:17. doi: 10.1186/1471-2105-12-17 (PMC3033801; doi:10.1186/1471-2105-12-17)
Supplement: Additional file 1 — Additional Tables S1, S2, S3 listing the power of different methods. Tables S1, S2 and S3 respectively summarizes the powers of different methods for three pathways "TNFR2 Signaling Pathway", "Fructose and mannose metabolism" and Cytokine-cytokine receptor interaction" in simulation studies. [file 1471-2105-12-17-S1.PDF]

**Table S1. Power of different methods for "TNFR2 Signaling Pathway" (pattern 1)**

| Relative Risk | FPC-AIC<br>Power (95% CI) P-value | FPC-BIC<br>Power (95% CI) P-value | FPC-FULL<br>Power (95% CI) P-value | AML<br>Power (95% CI) P-value | MSS<br>Power (95% CI) P-value | RTP_5<br>Power (95% CI) P-value | RTP_10<br>Power (95% CI) P-value |
|---|---|---|---|---|---|---|---|
| 1.00* ERRs | 0.94<br>(0.92,0.96)<br><0.001 | 0.90<br>(0.88,0.93)<br>NA | 0.86<br>(0.83,0.89)<br>0.007 | 0.72<br>(0.68,0.76)<br><0.001 | 0.81<br>(0.78,0.84)<br><0.001 | 0.85<br>(0.82,0.88)<br><0.001 | 0.83<br>(0.80,0.87)<br><0.001 |
| 0.99* ERRs | 0.83<br>(0.79,0.86)<br>0.001 | 0.78<br>(0.75,0.82)<br>NA | 0.75<br>(0.72,0.79)<br>0.085 | 0.59<br>(0.55,0.64)<br><0.001 | 0.70<br>(0.66,0.74)<br><0.001 | 0.74<br>(0.70,0.78)<br><0.001 | 0.69<br>(0.65,0.73)<br><0.001 |
| 0.98* ERRs | 0.70<br>(0.66,0.74)<br>0.003 | 0.65<br>(0.61,0.69)<br>NA | 0.61<br>(0.57,0.65)<br>0.028 | 0.43<br>(0.39,0.48)<br><0.001 | 0.54<br>(0.49,0.58)<br><0.001 | 0.55<br>(0.51,0.60)<br><0.001 | 0.52<br>(0.47,0.56)<br><0.001 |
| 0.97* ERRs | 0.57<br>(0.52,0.61)<br><0.001 | 0.50<br>(0.46,0.55)<br>NA | 0.49<br>(0.45,0.54)<br>0.633 | 0.29<br>(0.25,0.33)<br><0.001 | 0.40<br>(0.36,0.44)<br><0.001 | 0.42<br>(0.38,0.46)<br><0.001 | 0.37<br>(0.33,0.41)<br><0.001 |
| 0.96* ERRs | 0.44<br>(0.40,0.49)<br><0.001 | 0.37<br>(0.33,0.42)<br>NA | 0.35<br>(0.31,0.40)<br>0.291 | 0.21<br>(0.17,0.24)<br><0.001 | 0.29<br>(0.25,0.33)<br><0.001 | 0.32<br>(0.28,0.36)<br><0.001 | 0.27<br>(0.23,0.31)<br><0.001 |

**Note: For each method, we list the power, the 95% confidence interval of all estimated probabilities, the p-value of McNemar's test for the difference between FPC_BIC with any other methods. The power is calculated on the basis of 500 replicates at significance level 0.05.**

**Table S2. Power of different methods for “Fructose and mannose metabolism” (pattern 2)**

| Relative Risk | FPC-AIC<br>Power<br>(95% CI)<br>P-value | FPC-BIC<br>Power<br>(95% CI)<br>P-value | FPC-FULL<br>Power<br>(95% CI)<br>P-value | AML<br>Power<br>(95% CI)<br>P-value | MSS<br>Power<br>(95% CI)<br>P-value | RTP_5<br>Power<br>(95% CI)<br>P-value | RTP_10<br>Power<br>(95% CI)<br>P-value |
|---|---|---|---|---|---|---|---|
| 1.00* ERRs | 0.66<br>(0.62,0.71)<br>0.040 | 0.63<br>(0.58,0.67)<br>NA | 0.53<br>(0.49,0.58)<br><0.001 | 0.44<br>(0.40,0.49)<br><0.001 | 0.43<br>(0.38,0.47)<br><0.001 | 0.52<br>(0.48,0.57)<br><0.001 | 0.50<br>(0.46,0.55)<br><0.001 |
| 0.99* ERRs | 0.53<br>(0.48,0.57)<br>0.008 | 0.47<br>(0.43,0.52)<br>NA | 0.42<br>(0.37,0.46)<br>0.013 | 0.32<br>(0.28,0.36)<br><0.001 | 0.30<br>(0.26,0.34)<br><0.001 | 0.37<br>(0.33,0.42)<br><0.001 | 0.35<br>(0.31,0.39)<br><0.001 |
| 0.98* ERRs | 0.39<br>(0.35,0.44)<br>0.396 | 0.38<br>(0.33,0.42)<br>NA | 0.32<br>(0.28,0.36)<br>0.009 | 0.25<br>(0.21,0.29)<br><0.001 | 0.23<br>(0.19,0.27)<br><0.001 | 0.28<br>(0.24,0.32)<br><0.001 | 0.25<br>(0.21,0.28)<br><0.001 |
| 0.97* ERRs | 0.29<br>(0.25,0.33)<br>0.420 | 0.28<br>(0.24,0.32)<br>NA | 0.23<br>(0.20,0.27)<br>0.022 | 0.17<br>(0.13,0.20)<br><0.001 | 0.15<br>(0.12,0.18)<br><0.001 | 0.18<br>(0.15,0.21)<br><0.001 | 0.18<br>(0.15,0.21)<br><0.001 |
| 0.96* ERRs | 0.20<br>(0.18,0.24)<br>0.440 | 0.19<br>(0.15,0.22)<br>NA | 0.18<br>(0.14,0.21)<br>0.648 | 0.11<br>(0.08,0.14)<br><0.001 | 0.11<br>(0.09,0.14)<br><0.001 | 0.12<br>(0.09,0.15)<br><0.001 | 0.11<br>(0.08,0.14)<br><0.001 |

**Table S3. Power of different methods for “Cytokine-cytokine receptor interaction” (pattern 3)**

| Relative Risk | FPC-AIC<br>Power (95% CI) P-value | FPC-BIC<br>Power (95% CI) P-value | FPC-FULL<br>Power (95% CI) P-value | AML<br>Power (95% CI) P-value | MSS<br>Power (95% CI) P-value | RTP_5<br>Power (95% CI) P-value | RTP_10<br>Power (95% CI) P-value |
|---|---|---|---|---|---|---|---|
| 1.00* ERRs | 0.97 (0.96,0.99) 0.194 | 0.96 (0.94,0.97) NA | 0.92 (0.90,0.95) 0.016 | 0.93 (0.91,0.95) 0.069 | 0.80 (0.77,0.84) <0.001 | 0.88 (0.85,0.91) <0.001 | 0.90 (0.88,0.93) <0.001 |
| 0.99* ERRs | 0.88 (0.86,0.91) 0.741 | 0.88 (0.85,0.91) NA | 0.79 (0.75,0.82) <0.001 | 0.79 (0.76,0.83) <0.001 | 0.66 (0.62,0.70) <0.001 | 0.74 (0.70,0.78) <0.001 | 0.78 (0.74,0.82) <0.001 |
| 0.98* ERRs | 0.76 (0.72,0.80) 0.111 | 0.79 (0.76,0.83) NA | 0.62 (0.58,0.67) <0.001 | 0.59 (0.55,0.63) <0.001 | 0.49 (0.45,0.53) <0.001 | 0.57 (0.53,0.62) <0.001 | 0.59 (0.55,0.64) <0.001 |
| 0.97* ERRs | 0.58 (0.54,0.62) 0.458 | 0.60 (0.55,0.64) NA | 0.45 (0.41,0.50) <0.001 | 0.38 (0.34,0.43) <0.001 | 0.31 (0.27,0.35) <0.001 | 0.38 (0.34,0.43) <0.001 | 0.40 (0.46,0.45) <0.001 |
| 0.96* ERRs | 0.44 (0.40,0.49) 0.057 | 0.48 (0.44,0.53) NA | 0.34 (0.30,0.38) <0.001 | 0.24 (0.20,0.28) <0.001 | 0.25 (0.21,0.29) <0.001 | 0.28 (0.24,0.32) <0.001 | 0.26 (0.22,0.29) <0.001 |
