# Supplementary material for: Pathway-based analysis using reduced gene subsets in genome-wide association studies
Source: BMC Bioinformatics. 2011 Jan 12;12:17. doi: 10.1186/1471-2105-12-17 (PMC3033801; doi:10.1186/1471-2105-12-17)
Supplement: Additional file 2 — Additional Table S4 listing the top 10 associated genes in GAIN data set. Table S4 lists the top 10 genes associated in psoriasis GAIN data set by using the FPC-based gene level test. [file 1471-2105-12-17-S2.PDF]

Table S4. The top 10 genes in psoriasis GAIN data set

Chr	Gene	# of SNPs	FPC-based p-value
1	100130497-LOC100130497	1	2.40E-07
5	3596-IL13	5	3.04E-06
5	10111-RAD50	3	3.36E-06
12	6773-STAT2	2	1.61E-05
3	10354-HMG1L5	5	2.11E-05
12	51561-IL23A	1	2.37E-05
6	7128-TNFAIP3	7	2.58E-05
12	93058-COQ10A	1	2.99E-05
12	1431-CS	3	3.20E-05
20	27296-TP53TG5	2	5.03E-05
9	7248-TSC1	15	5.07E-05

Note: The column "# of SNPs" denotes the number of SNPs in each gene. For each gene, we build a logistic regression model using its gene-based FPC score and use the LR test to perform the gene-level analysis. The p-values for the top 10 genes are listed in the last column "FPC-based p-value".
